# Much More Than a Stroke: Emery-Dreifuss Muscular Dystrophy Type 2 Revealed by Ischemic Stroke

**DOI:** 10.7759/cureus.73815

**Published:** 2024-11-16

**Authors:** Francisco Gonçalves, Daniela Duarte, Filipa Reis, Alexandra Vaz, Ana Gomes

**Affiliations:** 1 Department of Internal Medicine, Centro Hospitalar Tondela-Viseu, Viseu, PRT; 2 Stroke Unit, Centro Hospitalar Tondela-Viseu, Viseu, PRT

**Keywords:** acute cerebral ischemic stroke, emery-dreifuss muscular dystrophy, genetic myopathy, laminopathy, non-ischemic dilated cardiomyopathy

## Abstract

Emery-Dreifuss muscular dystrophy type 2 (EDMD2) is a rare autosomal dominant neuromuscular disorder caused by LMNA gene mutations and characterized by progressive skeletal muscle weakness and significant cardiac involvement.

We report the case of a 45-year-old woman who presented with sudden-onset, left-sided hemiparesis and dysarthria. Initial imaging was unremarkable, and symptoms transiently improved, suggesting a transient ischemic attack. However recurrent deficits led to the identification of right middle cerebral artery occlusion and new-onset atrial fibrillation. Mechanical thrombectomy was successfully performed.

Subsequent cardiac evaluation revealed dilated cardiomyopathy with moderately depressed systolic function and segmental wall motion abnormalities, although coronary arteries were normal. Cardiac magnetic resonance imaging demonstrated myocardial fibrosis with late gadolinium enhancement in the subendocardial and mid-wall regions, suggestive of genetic cardiomyopathy.

A neurological examination noted lordotic posture, waddling gait, positive Gowers' sign, generalized proximal muscle atrophy, and flaccid hyporeflexic tetraparesis. Electromyography confirmed a proximal myopathy. The patient reported limb weakness since her twenties. Family history was significant for similar neuromuscular and cardiac symptoms.

Genetic testing identified a heterozygous LMNA missense variant (ClinVar ID: 804298), confirming the diagnosis of EDMD2. Despite the implantation of a cardiac resynchronization therapy defibrillator and optimal medical management, she experienced recurrent ventricular tachyarrhythmias, necessitating listing for heart transplantation.

This case highlights the diagnostic challenges of EDMD2, particularly when the initial presentation is ischemic stroke, a rare manifestation of this genetic myopathy. It underscores the importance of a multidisciplinary approach for early diagnosis and management to improve the outcomes of this rare but impactful disorder.

## Introduction

Emery-Dreifuss muscular dystrophy (EDMD) is a rare, progressive genetic disorder characterized by a triad of early-onset joint contractures, progressive skeletal muscle weakness and atrophy, and cardiac involvement manifesting as conduction defects, arrhythmias, and cardiomyopathy [1-3]. The cardiac complications associated with EDMD are particularly significant, often leading to sudden cardiac death [4,5].

EDMD exhibits genetic heterogeneity with three primary inheritance patterns: X-linked recessive (EDMD1), autosomal dominant (EDMD2), and autosomal recessive (EDMD3) [2,6]. Both the autosomal dominant EDMD2 and the rare autosomal recessive EDMD3 are linked to pathogenic variants in the LMNA gene located on chromosome 1q21.2 [1,6]. This gene encodes lamin A and lamin C, two A-type lamins integral to the nuclear envelope's structural integrity and involved in essential cellular functions [7].

The clinical manifestations of EDMD are generally similar across the different genetic forms, typically presenting in the first or second decade of life. However, there is significant inter- and intrafamilial variability regarding the age at onset, severity, and progression of symptoms [8]. Muscle weakness usually begins in the upper limbs, affecting the biceps brachii and triceps brachii muscles, with relative preservation of the deltoid muscles. As the disease progresses, weakness extends to the distal lower limbs, notably causing atrophy of the peroneal muscles and resulting in gait disturbances. The myopathy tends to progress slowly during the first three decades of life but accelerates thereafter [3,9].

Cardiac involvement is a hallmark of EDMD and is more aggressive in EDMD2 [4,5]. Patients frequently develop dilated cardiomyopathy (DCM), characterized by left ventricular systolic dysfunction and enlargement of the ventricular chambers in the absence of abnormal loading conditions such as hypertension, valvular heart disease, or coronary artery disease [4,10]. Ventricular arrhythmias often occur earlier in EDMD2, contributing to the development of DCM and leading to progressive heart failure and an increased risk of arrhythmia-related sudden death [4,5].

This case elucidates the diagnostic challenges and clinical complexities of EDMD2, underscored by a patient who presented with an ischemic stroke, a rare initial manifestation of this genetic myopathy [11].

This case report was previously presented as a poster at the 20th European Congress of Internal Medicine, held in Málaga, Spain, from June 9 to 11, 2022.

## Case presentation

A 45-year-old woman with no significant medical history and not on any regular medications presented to the emergency department with sudden-onset, left-sided hemiparesis and dysarthria of two hours' duration. On initial examination, her neurological deficits had markedly improved, with resolution of dysarthria and only a slight decrease in dexterity of the left hand.

A non-contrast cranial computed tomography (CT) scan revealed no evidence of acute ischemic or hemorrhagic lesions. Given the transient nature of her symptoms and unremarkable imaging, a provisional diagnosis of a transient ischemic attack (TIA) was made. She was admitted for close neurological observation.

**Figure 1:**
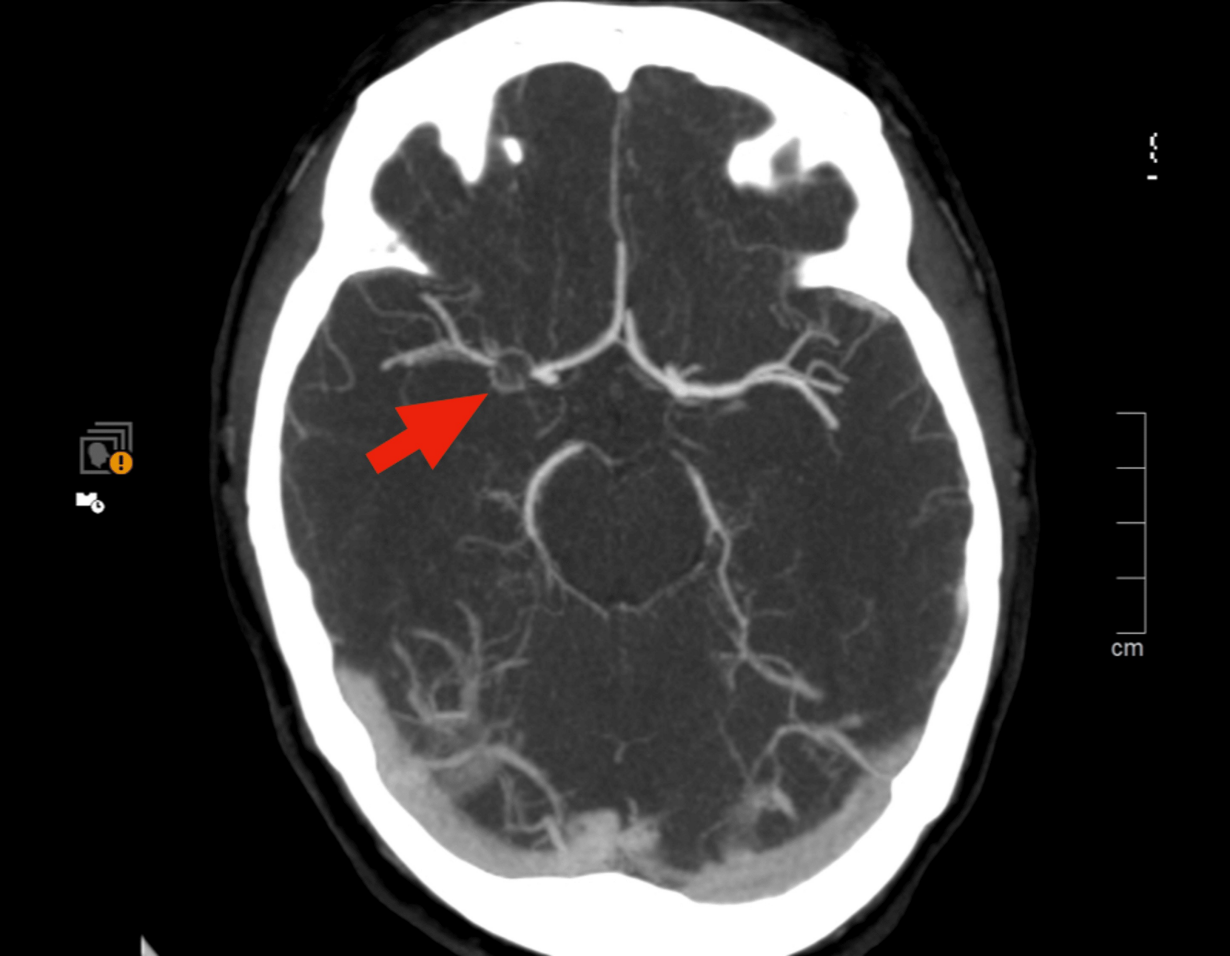
Cranial CT angiography shows a stop sign in the M1 segment of the right middle cerebral artery

During the observation period, the patient experienced a recurrence of neurological deficits, including central left facial paralysis and decreased strength in the ipsilateral upper limb. An emergent cranial CT angiography demonstrated a subtle stop sign in the M1 segment of the right middle cerebral artery (MCA) ( Figure 1).

The case was promptly discussed with the neuroradiology team, who interpreted the findings as suggestive of a moyamoya-like vasculopathy rather than an occlusive thrombus, thereby precluding immediate endovascular intervention. The patient was admitted for further monitoring and scheduled for cranial magnetic resonance imaging (MRI) with angiography.

In the early hours of the following day, the in-hospital stroke team was activated due to the sudden onset of left-sided hemiplegia, hypoesthesia, central facial paralysis, and dysarthria. The patient remained hemodynamically stable but was noted to have an irregular pulse. Electrocardiography (ECG) revealed new-onset atrial fibrillation.

A repeat cranial CT angiography showed a right striatocapsular hypodensity causing slight adjacent ventricular wall molding, indicative of a recent ischemic infarct. The M1 segment of the right MCA remained occluded. After re-evaluation by the neuroradiology team, the occlusion was reclassified as thromboembolic in origin. Mechanical thrombectomy was promptly performed, achieving successful recanalization with a Thrombolysis in Cerebral Infarction score of 2b. No procedural complications occurred.

**Figure 2:**
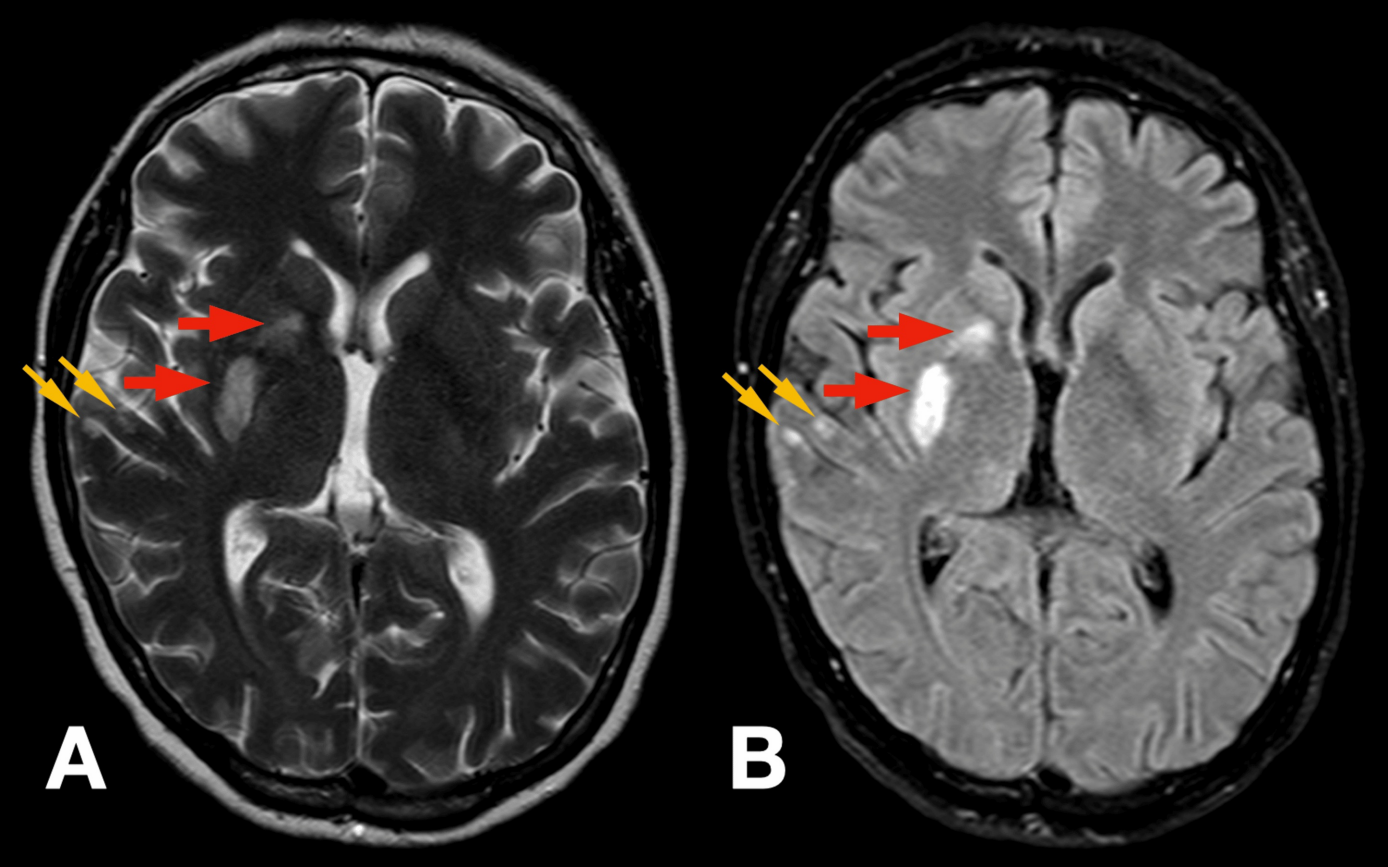
Cranial MRI demonstrating multi-focal ischemic stroke T2-weighted image (Panel A) and fluid-attenuated inversion recovery (FLAIR) image (Panel B) demonstrate hyperintensity in the corona radiata and striatocapsular area (red arrows) and multiple cortical foci within the territory of the right middle cerebral artery (orange arrows) as a result of irreversible acute ischemic brain damage.

**Figure 3:**
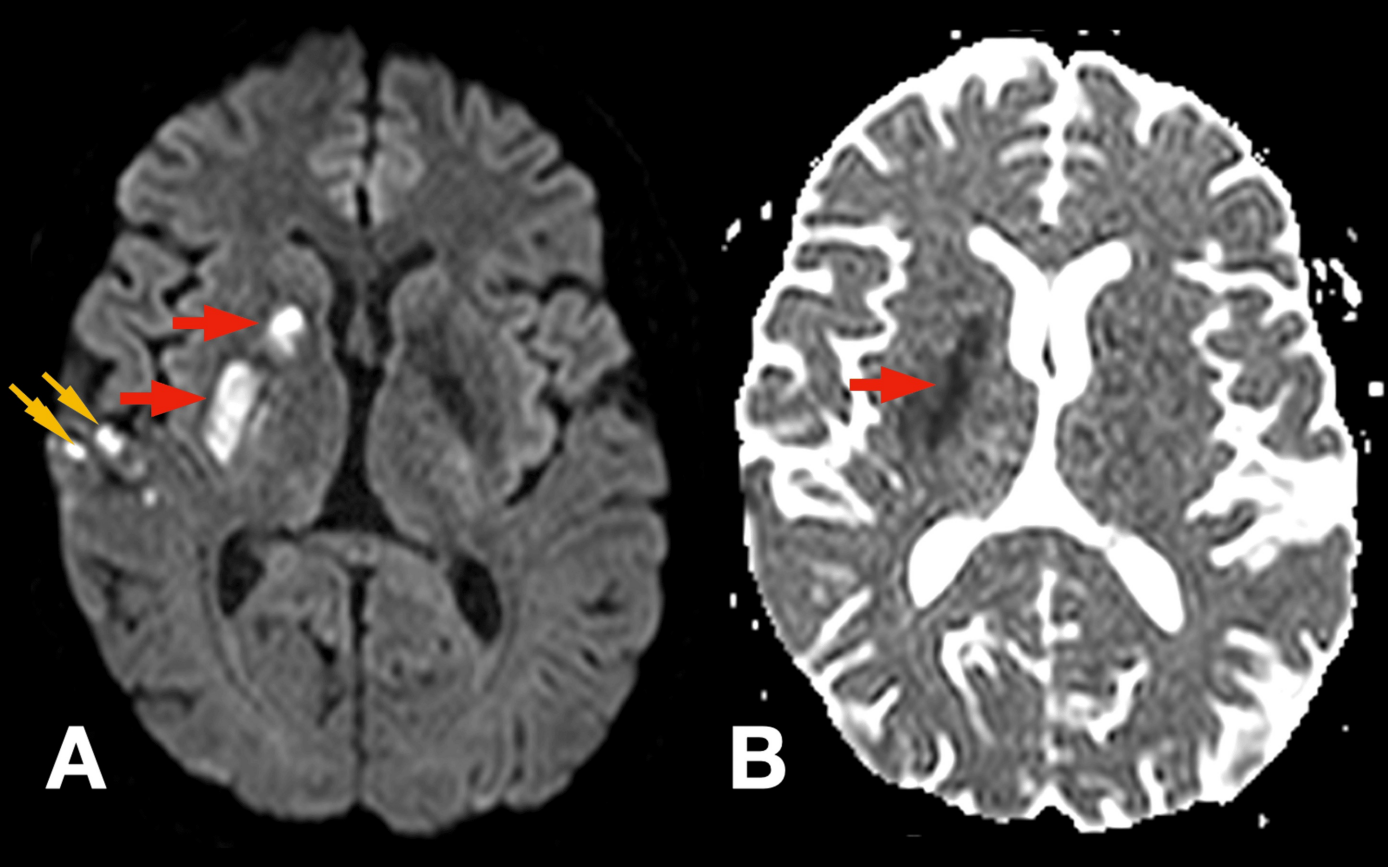
Cranial MRI demonstrating multi-focal ischemic stroke The lesions appear as hyperintense areas on diffusion-weighted image (DWI) (Panel A) and as correlative hypointense areas on apparent diffusion coefficient (ADC) (Panel B), in a pattern of restricted diffusion.

Post-procedure, the patient exhibited significant neurological improvement. Muscle strength in the left upper and lower limbs improved to grade 4/5 on the Medical Research Council (MRC) scale, and dysarthria resolved. Mild residual left central facial asymmetry persisted. The National Institutes of Health Stroke Scale (NIHSS) score decreased from 12 at presentation to 2 post-thrombectomy. After stabilization, a cranial MRI was performed, which demonstrated an acute ischemic lesion in the right striatocapsular and corona radiata regions, as well as multiple cortical foci within the territory of the right middle cerebral artery (Figures 2, 3). 

During her hospitalization in the stroke unit, an etiological workup for a cardioembolic source was initiated, given the new diagnosis of atrial fibrillation in a relatively young patient without significant medical history. Transthoracic echocardiography (TTE) revealed dilation of all cardiac chambers, with pronounced left ventricular enlargement, severely dilated left ventricle (left ventricular end-diastolic diameter of 65 mm) with moderately depressed systolic function (left ventricular ejection fraction (LVEF) of 37%), segmental wall motion abnormalities in the inferior and inferolateral walls, and moderate functional mitral regurgitation due to annular dilation.

**Figure 4:**
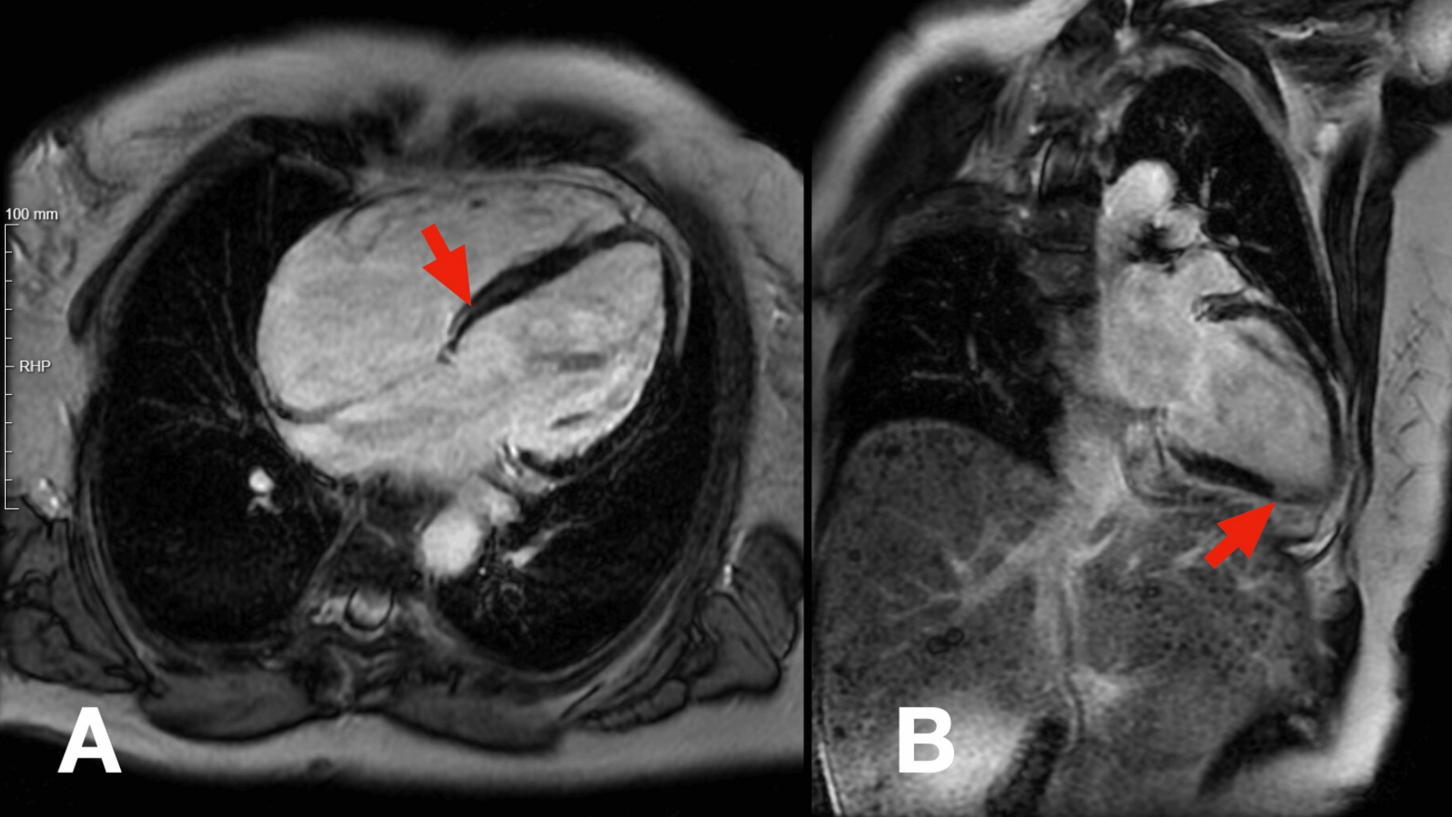
Cardiac MRI with late gadolinium enhancement Red arrows in panels A and B show moderate myocardial fibrosis in a mixed pattern, involving both the subendocardial and mid-wall regions.

A Cardiology consultation was obtained. Coronary angiography demonstrated a left-dominant coronary circulation without significant stenoses or atherosclerotic lesions. Cardiac MRI corroborated the findings of dilated cardiomyopathy, showing global hypokinesia and an LVEF of 35%. Late gadolinium enhancement (LGE) imaging revealed moderate myocardial fibrosis in a mixed pattern, involving both ischemic (subendocardial) and non-ischemic (mid-wall) regions, particularly in the lateral wall (Figure 4), suggestive of a genetic cardiomyopathy.

Upon mobilization, further neurological examination revealed signs consistent with a neuromuscular disorder. The patient exhibited a lordotic posture and a waddling, myopathic gait. Rising from a low chair required the use of her hands to push off her thighs (Gowers' maneuver). Generalized muscle atrophy was evident, predominantly affecting the proximal muscles of the scapular and pelvic girdles. Mild atrophy of the temporalis muscles and thenar eminences was also noted. The extended-arm test showed slight drooping and pronation of the left upper limb. Muscle strength testing revealed flaccid, hyporeflexic tetraparesis, graded as 3+/5 proximally and 4/5 distally, relatively symmetrical aside from the mild left hemiparesis due to the recent stroke.

Based on the clinical presentation of non-ischemic dilated cardiomyopathy with depressed systolic function and proximal muscle weakness with a myopathic gait and positive Gowers' sign, a primary myopathy affecting both skeletal and cardiac muscle was suspected.

With the support of Neurology, electromyography (EMG) was performed, confirming a myopathy of moderate severity with predominant proximal involvement and minimal signs of muscle fiber necrosis (Table 1). Laboratory studies showed borderline elevations of muscle injury markers, with a serum creatine kinase (CK) level of 223 U/L (reference range: 30-200 U/L).

**Table 1:** Electromyographic assessment of spontaneous and voluntary muscle activity in right-sided muscles Electromyographic findings from the right posterior deltoid, biceps brachii, first dorsal interosseous, rectus femoris, and tibialis anterior muscles show polyphasic motor unit potentials and early recruitment patterns, indicating a myopathic process. Occasional fasciculations (1+) are present while the absence of fibrillation potentials and positive sharp waves reduces the likelihood of acute denervation.

		Spontaneous Activity			Voluntary Activity				
Muscle	Interpretation	Fibrillation potentials	Positive sharp waves	Fasciculations	Amplitude of motor unit potentials	Duration of motor unit potentials	Polyphasic potentials	Insertional potential	Recruitment pattern
Right Deltoideus Posterior	Myopathy	0	0	1+	-	-	+	+	Early
Right Biceps	Myopathy	0	0		-	-	+	+	Early
Right Interosseous Dorsalis	Myopathy	0	0		-	-	+	+	Early
Right Rectus Femoris	Myopathy	0	0	1+	-	-	+	+	Early
Right Tibialis Anterior	Myopathy	0	0		-	-	+	+	Early

A detailed anamnesis revealed that the patient had experienced limb muscle weakness since her early 20s, including difficulty climbing stairs and raising her arms above her head. She denied myotonia, dysphonia, or dysphagia. A thorough family history disclosed that her father and sisters had similar symptoms of proximal muscle weakness, atypical gait, and cardiac disease. Notably, her father (now deceased), one sister, and two maternal nephews had pacemaker defibrillators implanted at a young age. Another older sister had been evaluated for Brugada syndrome, although genetic testing was negative.

Genetic testing was pursued. Next-generation sequencing of a myopathy gene panel identified a heterozygous missense variant in the LMNA gene (NM_170707.3:c.80C>T p.(Thr27Ile), located in exon 1 of 12. This variant is classified as likely pathogenic (ClinVar ID: 804298) and has been reported in patients with laminopathies presenting with both skeletal muscle involvement and cardiac abnormalities. Pathogenic variants in the LMNA gene cause, among other conditions, autosomal dominant Emery-Dreifuss muscular dystrophy type 2 (EDMD2; MIM 181350), and the Slovenian type hand-heart syndrome (MIM 610140), as well as autosomal recessive Emery-Dreifuss muscular dystrophy type 3 (EDMD3; MIM 616516). Based on the clinical findings and family history, the most probable diagnosis was EDMD2. Immediate family members were referred for genetic counseling and cascade genetic testing.

During subsequent follow-up in the cardiology outpatient clinic, the patient underwent implantation of a cardiac resynchronization therapy defibrillator (CRT-D) due to the diagnosis of non-ischemic dilated cardiomyopathy associated with the LMNA mutation.

Despite optimal medical and device therapy, the patient experienced multiple episodes of ventricular tachyarrhythmias necessitating defibrillator shocks, culminating in admission to the coronary care unit due to an arrhythmic storm. Given her refractory ventricular tachycardia unamenable to catheter ablation and progressive heart failure, she was evaluated and listed for heart transplantation.

## Discussion

This case highlights the diagnostic challenges and complexity of managing a patient with EDMD2, particularly when presenting atypically with an ischemic stroke. The initial presentation suggested a cardioembolic event secondary to atrial fibrillation, a common cause of ischemic strokes in the general population. However, the occurrence of atrial fibrillation and stroke in a relatively young woman without significant medical history prompted a comprehensive evaluation for underlying cardiomyopathies and genetic conditions.

Cardiac imaging revealed a non-ischemic DCM with a moderately depressed left ventricular ejection fraction. The presence of segmental wall motion abnormalities and late gadolinium enhancement on cardiac MRI suggested a myocardial disease of genetic etiology [10]. Concurrently, neurological examination uncovered proximal muscle weakness with a myopathic gait and positive Gowers' sign, indicative of a neuromuscular disorder [3,9]. Electromyography confirmed a myopathy of moderate severity.

The detailed family history of proximal muscle weakness and early-onset cardiac disease requiring pacemaker-defibrillator implantation in several relatives strengthened the suspicion of a hereditary condition. Genetic testing identified a heterozygous pathogenic variant in the LMNA gene, confirming the diagnosis of EDMD2 [1,12].

EDMD2 is characterized by early-onset joint contractures, progressive muscle weakness and wasting, and significant cardiac involvement, including conduction defects, arrhythmias, and DCM [1-3]. The cardiac manifestations are often more aggressive in EDMD2 compared to other forms of EDMD, with ventricular arrhythmias occurring earlier and contributing to the development of DCM and heart failure [4,5].

Patients with EDMD, irrespective of the genetic subtype, are at increased risk of thromboembolic complications, particularly cerebral strokes, which tend to occur at a younger age than in the general population and can exacerbate the disability caused by muscular dystrophy [13,14]. Atrial fibrillation, as observed in this patient, is a known complication in EDMD2 and significantly elevates the risk of cardioembolic stroke.

This case illustrates that ischemic stroke can be an initial manifestation of EDMD2, albeit rare. According to the literature, in a small fraction of patients with EDMD, cerebral stroke has been the presenting feature leading to diagnosis [11]. Therefore, clinicians should maintain a high index of suspicion for underlying genetic myopathies in young patients presenting with cerebrovascular events and cardiac abnormalities.

The management of EDMD2 is challenging due to the multisystem involvement and progressive nature of the disease. Implantation of a CRT-D was crucial for the patient's survival, as she experienced recurrent episodes of ventricular arrhythmias requiring defibrillator shocks [4,5]. Despite these interventions, her condition progressed to refractory ventricular tachycardia and advanced heart failure, necessitating listing for heart transplantation [4,5].

Early diagnosis and intervention are essential to improve outcomes in patients with EDMD2. Genetic counseling and family screening are imperative due to the autosomal dominant inheritance pattern and the potential for severe cardiac complications at a young age. Early identification of at-risk relatives allows for surveillance and timely intervention, potentially reducing morbidity and mortality [15].

## Conclusions

This case underscores the critical importance of considering genetic myopathies, specifically EDMD2, in the differential diagnosis of young patients presenting with cerebrovascular events and cardiac abnormalities. The patient's initial presentation with an ischemic stroke secondary to new-onset atrial fibrillation illustrates the atypical manifestations of EDMD2. The subsequent identification of non-ischemic DCM with reduced systolic function, alongside proximal muscle weakness and a myopathic gait, prompted a comprehensive evaluation that established the diagnosis. Confirmation of a pathogenic variant in the LMNA gene was pivotal, highlighting the necessity of genetic testing.

A multidisciplinary approach involving internal medicine, neurology, cardiology, and genetics is essential to optimize patient outcomes. This case advocates for heightened clinical awareness of EDMD2 to facilitate early diagnosis, guide management strategies, and improve prognosis in this rare but impactful disorder.
